# Integrating arts and Environmental Education in museums: an evaluative case study of the EcoArt program

**DOI:** 10.3389/fpsyg.2026.1917355

**Published:** 2026-09-03

**Authors:** Fernando Echarri, María del Mar García-Echeverria, Teresa Barrio

**Affiliations:** 1 University of Navarra Museum, University of Navarra, Pamplona, Spain; 2 Coordinator of Educational Programs at Anthesis for the Commonwealth of the Pamplona Region, Mancoeduca, Pamplona/Iruña, Spain

**Keywords:** art-based experiences, Arts Integration, Deep Ecology, eco-psychology, Environmental Education, nature connection

## Abstract

Environmental challenges require educational approaches that foster ecological consciousness through interdisciplinary learning experiences. This article examines how Arts Integration and Environmental Education, grounded in the principles of Deep Ecology, can be developed within the context of an art museum. Rather than treating art and nature as separate domains, the study explores their complementary potential to cultivate meaningful engagement with ecological issues through aesthetic experience, creative inquiry, and critical reflection. In this case, art museums are conceptualised as educational environments where artistic interpretation and environmental awareness converge, enabling learners to establish deeper relationships with both cultural and natural heritage. Drawing on the theoretical foundations of Deep Ecology, Environmental Education, museum education, and Arts Integration, the article analyses the educational framework of the EcoArt program as a case study. The findings suggest that integrating artistic and ecological perspectives may strengthens ecological consciousness, and promotes commitments toward environmental sustainability. The study argues that art museum education can play a significant role in addressing contemporary environmental challenges by creating interdisciplinary learning experiences that connect aesthetic engagement with ecological responsibility.

## 1 Introduction

In recent decades, environmental degradation has become one of the greatest global challenges facing humanity. Beyond its ecological consequences, the environmental crisis also represents an educational challenge, as addressing it requires profound changes in the ways people understand, experience and relate to the natural world (Palmer, 1998; Novo, 2006). Consequently, Environmental Education (EE) has progressively evolved from approaches centred primarily on ecological knowledge toward broader perspectives that seek to foster environmental awareness, responsibility and active participation in sustainability (Kollmuss and Agyeman, 2002). Given the complexity of contemporary environmental problems, this educational response increasingly calls for interdisciplinary approaches (Novo, 2006) capable of integrating different forms of knowledge, experience and engagement.

From this perspective, environmental learning should not only enhance ecological knowledge but also strengthen people’s capacity to respond to environmental challenges. Brown and Westaway (2011) identify agency as a central dimension in understanding how individuals and communities adapt to environmental change, linking it to human wellbeing, resilience and capacity for action. Educational programs may therefore create learning experiences that help participants recognise their relationship with the natural world while developing the confidence, responsibility and commitment needed to engage in environmental action. This perspective is consistent with Deep Ecology (Næss, 1989), which challenges anthropocentric interpretations of the human–nature relationship (Beery et al., 2024) and proposes a broader ecological Self in which human identity and wellbeing are understood in relation to the more-than-human world (Leopold, 1966; Abram, 1996; Howell et al., 2013).

Arts Integration offers a pedagogical framework for connecting artistic processes with learning in other disciplines through creativity, inquiry, interpretation and aesthetic engagement (Burnaford, 2007; Silverstein and Layne, 2010; Goering et al., 2023). Rather than treating the arts as complementary or motivational resources, this approach recognises artistic practice as a legitimate mode of inquiry and knowledge construction (Bresler, 1995; Burnaford et al., 2001). Within EE, Arts Integration may facilitate educational experiences that engage learners cognitively, emotionally and experientially with environmental issues (Inwood, 2008, 2010; Inwood and Kennedy, 2020), encouraging them not only to understand ecological problems but also to interpret and respond to them in personally meaningful ways.

Art museums provide particularly valuable contexts for developing these interdisciplinary learning experiences. Museum learning has long been described as experiential, interpretative and constructivist, enabling visitors to connect objects with prior knowledge, emotions and personal experience (Hein, 1998; Hooper-Greenhill, 2007; Falk, 2009). Within this context, direct engagement with artworks can foster aesthetic experiences in which perception, emotion and reflection become interconnected (Dewey, 1934; Burnham and Kai-Kee, 2011). Consequently, artworks may function as mediating resources for exploring environmental relationships, sustainability and human responsibility, extending educational opportunities beyond the traditional boundaries of art education.

Although substantial research has examined Arts Integration (Bresler, 1995; Burnaford, 2007; Marshall, 2015; Green et al., 2018), Environmental Education (Novo, 2006; Graham, 2007; Inwood, 2008, 2010; Inwood and Kennedy, 2020) and museum learning (Hein, 1998; Hooper-Greenhill, 2007; Falk, 2009), comparatively few empirical studies have explored programs that intentionally integrate these three educational traditions within a single educational framework, particularly when such integration is explicitly informed by the principles of Deep Ecology. Examining these initiatives may contribute to understanding how the aesthetic and experiential potential of art museums can support environmental learning, awareness and agency.

To address this gap, the present study reports an evaluative case study of the EcoArt program, developed collaboratively by environmental educators and museum educators. EcoArt is situated at the intersection of three complementary dimensions: Deep Ecology constitutes its philosophical foundation, Arts Integration its pedagogical approach, and the art museum the experiential context in which environmental learning takes place. Rather than evaluating behavioural or wellbeing outcomes, this study examines teachers’ perceptions of the program’s educational value, identifying its perceived strengths and limitations while exploring its potential contribution to Environmental Education in art museums.

## 2 Theoretical framework

### 2.1 Environmental Education and the need for interdisciplinary approaches

Environmental degradation is increasingly recognised as a complex phenomenon that cannot be adequately understood from a single disciplinary perspective (Palmer, 1998). Ecological problems are simultaneously environmental, social, cultural, ethical (Caduto, 1985), political and educational (Novo, 2006). Consequently, EE has progressively evolved from approaches centred primarily on the transmission of ecological knowledge toward educational perspectives that seek to develop environmental awareness, critical reflection, participation and responsible action (Palmer, 1998).

Within this evolution, one of the principal challenges facing EE has been overcoming the persistent gap between environmental knowledge and pro-environmental behaviour (Kollmuss and Agyeman, 2002). Numerous educational programs have demonstrated that increasing ecological knowledge does not necessarily translate into greater environmental commitment or action. This situation has encouraged researchers to explore educational approaches capable of integrating cognitive, emotional, ethical and experiential dimensions of learning.

From this perspective, recent work has highlighted the importance of environmental agency, understood as people’s perceived capacity to contribute actively to environmental improvement. Brown and Westaway (2011) argue that agency constitutes a fundamental dimension of resilience and wellbeing because it enables individuals and communities to respond creatively to environmental challenges. Accordingly, EE should not only communicate scientific knowledge but also create educational experiences that strengthen learners’ capacity to recognise themselves as active participants in environmental care.

Such educational goals require interdisciplinary pedagogies capable of connecting scientific understanding with ethical reflection, emotional engagement and lived experience. It is within this context that the present study is situated.

### 2.2 Deep Ecology as the environmental philosophy

Among the different philosophical perspectives that have influenced EE, Deep Ecology offers a particularly comprehensive understanding of the relationship between human beings and the natural world (Næss, 1989). Rather than considering nature primarily as a resource for human use, Deep Ecology proposes an ecological worldview in which all living beings possess intrinsic value and human identity is understood as fundamentally embedded within ecological relationships.

From this perspective, environmental problems cannot be explained exclusively by technological deficiencies or insufficient scientific knowledge. They also reflect a progressive weakening of the relationship between people and the more-than-human world (Leopold, 1966; Abram, 1996). Consequently, environmental learning involves not only acquiring ecological concepts but also developing new ways of perceiving, experiencing and understanding our place within ecological systems.

This conception resonates with ecopsychology, which has consistently shown that meaningful contact with nature contributes to psychological wellbeing, cognitive restoration, emotional balance and life satisfaction (Roszak, 1995; Bowler et al., 2010; Fisher, 2013; Hartig et al., 2014; Capaldi et al., 2014; Moyers et al., 2024). From this perspective, environmental awareness, environmental agency and wellbeing should not be understood as independent educational outcomes but as interconnected dimensions of a broader ecological relationship.

Deep Ecology therefore provides the philosophical foundation of the EcoArt program. Rather than treating EE as the transmission of information about ecological problems, the program seeks to encourage participants to recognise the interdependence between human beings and the natural world, promoting reflection on environmental responsibility through aesthetic and experiential learning.

### 2.3 Arts Integration as the pedagogical framework

If Deep Ecology provides the environmental philosophy underlying the program, Arts Integration constitutes its pedagogical framework.

Arts Integration has emerged as an interdisciplinary educational approach that intentionally combines learning in the arts with learning in other academic disciplines through inquiry, creativity and aesthetic experience (Silverstein and Layne, 2010; Burnaford, 2007; Goering et al., 2023). Rather than treating artistic activities as complementary resources for motivating students, Arts Integration conceives artistic processes as legitimate forms of knowledge construction through which learners investigate complex questions from multiple perspectives.

This educational perspective draws upon constructivist theories of learning (Ausubel, 1968; Novak, 1976) and Dewey’s conception of education as experience (1934), according to which learning emerges through meaningful interaction between individuals and their environment. Within Arts Integration, aesthetic experience (Falk, 2009) becomes a mode of inquiry that encourages observation, interpretation, imagination, dialogue and reflection. Eisner (2002) similarly argued that the arts cultivate forms of thinking frequently neglected within conventional education, including perceptual awareness, interpretative judgement and the capacity to tolerate ambiguity. Greene (1995) further emphasized the role of aesthetic encounters in expanding learners’ capacity to question habitual ways of seeing the world.

These characteristics make Arts Integration particularly relevant for EE. Environmental issues rarely involve simple or predetermined solutions; instead, they require learners to interpret complex ecological relationships while simultaneously considering ethical, cultural and social dimensions. Previous studies have demonstrated that integrating artistic processes into EE can strengthen environmental awareness, emotional engagement and critical reflection (Inwood, 2008, 2010; Inwood and Kennedy, 2020).

In the present study, however, Arts Integration is understood as more than the combination of artistic and environmental contents. It constitutes the pedagogical process through which the philosophical principles of Deep Ecology are translated into meaningful educational experiences. Artistic interpretation, aesthetic perception and creative participation become the educational means through which learners explore ecological relationships and construct environmental meaning.

### 2.4 The art museum as an experiential learning environment

The educational potential of Arts Integration is particularly enhanced when it takes place within museum environments. Museums have progressively evolved from institutions centered primarily on the preservation of collections toward educational environments that promote active participation, interpretation and meaning-making (Hein, 1998; Hooper-Greenhill, 2007; Falk, 2009). Contemporary museum education understands learning as a personal and socially situated process in which visitors construct meanings through interaction with artworks, previous experiences and dialogue with others (Hooper-Greenhill, 2007).

Within this framework, artworks function not merely as cultural objects but as mediators of aesthetic experience capable of stimulating perception, emotion and reflection. Dewey (1934) argued that aesthetic experience integrates perception, feeling and understanding that facilitate meaningful learning. Similarly, Burnham and Kai-Kee (2011) emphasize that dialogue around artworks promotes interpretation rather than passive reception of information.

Art museums therefore provide educational contexts where EE may extend beyond scientific explanation alone. Through aesthetic engagement, learners are invited to explore environmental questions symbolically, emotionally and ethically while connecting environmental concepts with their own experiences. Furthermore, recent museum research has highlighted the contribution of museum experiences to different dimensions of personal and social wellbeing (Chatterjee and Noble, 2013; Falk, 2021), reinforcing their educational relevance for programs seeking to connect environmental awareness, human flourishing and cultural participation.

### 2.5 An integrated conceptual framework for the EcoArt program

The theoretical perspectives presented above should not be understood as independent educational traditions but as complementary dimensions of a single educational model. As a result of this integration, a program called EcoArt was proposed. Within an art museum, specifically at the University of Navarra Museum (hereafter MUN) (Navarra, Spain). This is a contemporary art museum inaugurated in 2015. Within the EcoArt program, Deep Ecology provides the environmental philosophy that defines the educational aims of fostering awareness of the human–nature relationship and environmental responsibility. Arts Integration provides the pedagogical framework through which these aims are translated into interdisciplinary learning experiences based on artistic interpretation, creativity and aesthetic engagement. Finally, the art museum provides the experiential learning context in which these educational processes take place, allowing participants to engage directly with artworks that stimulate reflection on ecological relationships and environmental responsibility.

Accordingly, EcoArt is not conceived simply as an arts-based EE program. Rather, it represents an interdisciplinary educational model in which environmental philosophy, pedagogical methodology and museum learning converge to create meaningful educational experiences capable of strengthening environmental awareness and environmental agency. From this perspective, the originality of the program lies not only in integrating art and EE but also in articulating these three educational dimensions within a coherent conceptual framework that guides both the educational design of the program and its evaluation.

## 3 Research design

### 3.1 Study design

This study adopted an *evaluative case study design* to examine teachers’ perceptions of the educational value and implementation of the EcoArt program. The study was conceived as an evaluation of an educational program implemented in an authentic museum context. Evaluation research occupies an important place within educational research because it provides systematic evidence regarding the quality, feasibility and perceived educational value of programs before larger-scale effectiveness studies are undertaken (Rossi et al., 2019). In this sense, the present study sought to determine how teachers perceived the educational design of EcoArt program, identify its principal strengths, and collect suggestions for its future improvement.

The study also adopts the characteristics of a *case study*, since it investigates a contemporary educational intervention within its real-life context (Yin, 2018). The MUN constitutes a unique educational setting where museum education and EE are intentionally integrated through a common pedagogical proposal. Consequently, understanding teachers’ evaluations of this particular program may provide valuable insights for future interdisciplinary initiatives developed in museum environments.

Teachers were selected as the principal informants because they accompanied their students throughout the program, observed their participation during the different educational activities, and evaluated the intervention from their own professional experience. Their perspectives therefore provide an informed assessment of the educational coherence, implementation and perceived value of the program.

### 3.2 Research questions

To guide the evaluation, the study addressed the following research questions:

*RQ1*. How do teachers evaluate the overall educational quality of the EcoArt program?

*RQ2*. Which educational dimensions of the program are perceived by teachers as its principal strengths?

*RQ3*. What recommendations do teachers provide for improving future implementations of the program?

These questions are consistent with the evaluative nature of the study. They aim to understand teachers’ professional judgements regarding the educational quality, coherence and potential contribution of the program as an interdisciplinary initiative integrating EE, Arts Integration and museum education.

### 3.3 Participants

The participants were *17 primary and secondary school teachers* who accompanied their students during the implementation of the EcoArt program at the MUN. Teachers represented different educational levels and disciplinary backgrounds, providing a diversity of educational perspectives on the program.

Teachers participated voluntarily and completed the evaluation questionnaire immediately after the educational intervention. Prior to participation, they were informed about the objectives of the study and provided their informed consent. All procedures complied with the ethical standards established by the University of Navarra Research Ethics Committee.

### 3.4 Instrument

Program evaluation was conducted using a structured questionnaire specifically designed to examine teachers’ perceptions of the EcoArt program. The instrument combined quantitative and qualitative items in order to obtain a comprehensive evaluation of the educational intervention.

The quantitative section consisted of Likert-type items assessing different dimensions of the program, including its educational organisation, interdisciplinary integration, perceived educational value, students’ engagement and overall quality. In addition, teachers were invited to provide open-ended comments concerning the strengths of the program and suggestions for future improvement.

Combining quantitative ratings with qualitative comments allowed the study not only to obtain an overall assessment of teachers’ satisfaction but also to explore the educational aspects that participants considered particularly valuable and the improvements they recommended for subsequent implementations.

### 3.5 Data collection

Data were collected immediately after completion of the EcoArt program. Following the educational activities, participating teachers completed the evaluation questionnaire individually. Collecting the data immediately after the intervention ensured that teachers’ evaluations reflected their direct experience of the program while the educational activities remained recent.

Questionnaires were completed anonymously to encourage honest responses and minimise potential social desirability bias.

### 3.6 Data analysis

Quantitative data were analysed using descriptive statistics, including frequencies, percentages, means and standard deviations, in order to summarise teachers’ evaluations of the different dimensions of the program.

Responses to the open-ended questions were analysed qualitatively through an inductive thematic analysis. Teachers’ comments were examined repeatedly to identify recurring themes concerning the perceived educational strengths of the program and recommendations for its improvement. The integration of quantitative and qualitative evidence provided a richer understanding of teachers’ evaluations than either approach alone, allowing the numerical ratings to be interpreted alongside participants’ own explanations and reflections.

## 4 The EcoArt program: a Deep Ecology adventure in an art Museum

It is due to the possibility of generating such experiences and the need to continue proposing programs that combine Environmental Education and Arts Integration that a Deep Ecology program was proposed within an art museum. The two entities that designed the project are Mancoeduca (Navarra, Spain) and the University of Navarra Museum (hereafter MUN) (Navarra, Spain). Mancoeduca is a EE section of a public institution that brings together 50 villages with the main objective of improving EE of its inhabitants, with a very solid background, with more of 40 years of experience^1^. The MUN is a contemporary art museum inaugurated in 2015 that carries out various Arts Integration programs aimed at the school population. Both entities combined their strengths to establish a joint synergy.

As a result of this alliance during the 2025–2026 academic year, an innovative for its region program was designed aimed at the schoolchildren audience called “EcoArt: A Deep Ecology adventure in an Art Museum” (hereafter, EcoArt) The EcoArt program was designed as an interdisciplinary educational intervention integrating Deep Ecology, Arts Integration and museum education within a coherent educational framework. Thus, the program also consistently incorporate innovation, understood as a systematic and planned process of transformative actions aimed at improving teaching and learning processes. Specifically, three fundamental axes are addressed in order to transform our educational practices:

- Experiential education, which may foster more meaningful learning (Ausubel, 1968; Novak, 1976).
- Active learning methodologies, incorporating collaborative and participatory dynamics into sessions (Prince, 2004).
- Full inclusion, enhancing the program’s capacity to reach all people.

### 4.1 Learning objectives

The EcoArt program aims the following objectives:

- promote reflection on the human–nature relationship from the perspective of Deep Ecology;
- foster environmental awareness through aesthetic experience;
- strengthen environmental agency through creative participation.

### 4.2 Educational sequence

The program had a duration of 2 hours. The EcoArt program was organised as a progressive learning sequence in which each educational activity contributed to the development of environmental awareness through aesthetic experience. Rather than presenting artworks as isolated objects of observation, the program combined artistic interpretation, environmental reflection and creative participation within a coherent educational process. The sequence was structured into four interconnected phases. The instructional sequence of the different phases of the program and their timing are presented in Table 1. The methodological alignment of the different activities proposed in the educational program according to the instructional sequence is presented in Table 2.

**Table 1:** Didactic sequence of the various phases of the program and their timing.

Phase	Timing	Location
Introduction	5′	Workshop
Motivation	15′	Workshop
Research	50′	Exhibition galleries
Creative activity	35′	Workshop
Reflection	10′	Workshop
Closure and evaluation	5′	Workshop

**Table 2:** Methodological fit of the different activities of the program with their impact on wellbeing, artistic references and resources.

Didactic sequence of activities	Environmental Education intervention (Mancoeduca)	Intervention of art education (MUN)	Impact on wellbeing	Artistic references and resources
A1. Introduction (5 min)	Approach to the Person-Nature Link: The Self and the More Than Human World. Inner world and outer world	Museum context: Reality and art as making-special	Individual and social: Awareness of the self and other selves	Museum context
A2. Motivation (15 min)	Vital awareness: Reflection on the dependence on water and oxygen for life.	Mindfulness: Dynamics of visual “scanning” in pairs and use of masks for the inner gaze.	Individual: Stress reduction and promotion of body and eudaimonic awareness.	Fabric masks for introspection.
A3. Research 1 (10 min)	Original nature: The life cycle, the soil and plant elements such as the fern.	Detailed Observation: Encouraging a careful eye to distinguish unique elements in the composition.	Planet: Awareness of the ecological footprint and the value of the natural as opposed to the transformed.	Pahoehoe by Carlos Irijalba (photograph of the volcano on La Palma).
A4. Research 2 (10 min)	Deep Ecology: Equality between human beings and other natural beings; principle of non-exploitation.	Bond and Essence: Exploration of identity and human connection through portraiture.	Group: Strengthening empathy and the sense of belonging to the biotic community.	Armando and other portraits by Pierre Gonnord.
A5. Research 3 (10 min)	Biological cycles: Analysis of biodiversity, light and natural order in the face of entropy and anthropic interventions.	Geometry and Revaluation: Use of space and materials to question aesthetic prejudices.	Planet and group: Recognition of interconnectedness in complex systems.	Twilight by Irijalba; work by Palazuelo; Box of Tàpies.
A6. Creative Action (35 min)	Citizen commitment: Choice of 1 to 3 actions to reduce the impact (water, waste, transport).	I am an Ecoartist Workshop: Plastic creation to represent the personal link with nature.	Individual: Empowerment and a sense of agency to transform reality.	Cardboard, leaflets, triptychs or cubes (depending on level).
A7. Reflection and Closing (15 min)	Collective action: Symbolic watering of the fern with the water collected from all the “drops” of the group.	Communication: Sharing of works and commitments to the group.	Planet and group: Closing the circle of care and recognition of the transformative power of the whole.	Live fern and water sprayer.

#### 4.2.1 Phase 1. Introduction: Framing the environmental challenge

The program began with a brief introductory session in which environmental educators presented the main aims of the activity and introduced students to the environmental challenges addressed throughout the program. Rather than providing an exhaustive explanation of ecological problems, this initial phase sought to stimulate curiosity and prepare students for an interdisciplinary exploration of the relationship between human beings, nature and artistic expression. Students were also introduced to the principles of Deep Ecology that would underpin the subsequent activities, encouraging them to reflect upon the intrinsic value of nature and the interdependence between human beings and the more-than-human world.

#### 4.2.2 Phase 2. Aesthetic exploration of the artworks

The second phase took place in the museum galleries, was a visual thinking activity (Yenawine, 2013) where students engaged with a carefully selected group of artworks through guided observation and dialogue. Following an Arts Integration approach, museum educators encouraged participants to examine the artworks from multiple perspectives, fostering careful observation, interpretation, discussion and personal reflection. Rather than transmitting predetermined interpretations, the educational process invited students to construct meanings collectively while exploring environmental themes suggested by the artworks. Throughout this phase, aesthetic experience functioned as a catalyst for environmental reflection, allowing students to establish connections between artistic expression, ecological relationships and their own lived experiences.

#### 4.2.3 Phase 3. Creative inquiry and environmental reflection

Building upon the dialogue generated during the museum visit, students participated in creative activities designed to deepen their understanding of the environmental issues explored through the artworks. These activities encouraged participants to express ideas, emotions and reflections through artistic creation while simultaneously considering questions related to environmental responsibility, sustainability and the human–nature relationship. In this way, artistic production became not an end in itself but a means of constructing environmental meaning through active participation and commitments.

#### 4.2.4 Phase 4. Collective reflection and synthesis

The program concluded with a shared reflective session in which students discussed their experiences, exchanged interpretations and considered the educational significance of the activities. This final phase sought to integrate the different dimensions of the program by connecting aesthetic experience, environmental reflection and collaborative dialogue. Rather than evaluating artistic performance, emphasis was placed on encouraging participants to articulate the meanings they had constructed throughout the experience and to reflect upon the environmental awareness and agency.

### 4.3 Innovative aspects of the program and its activities

**Figure 1:**
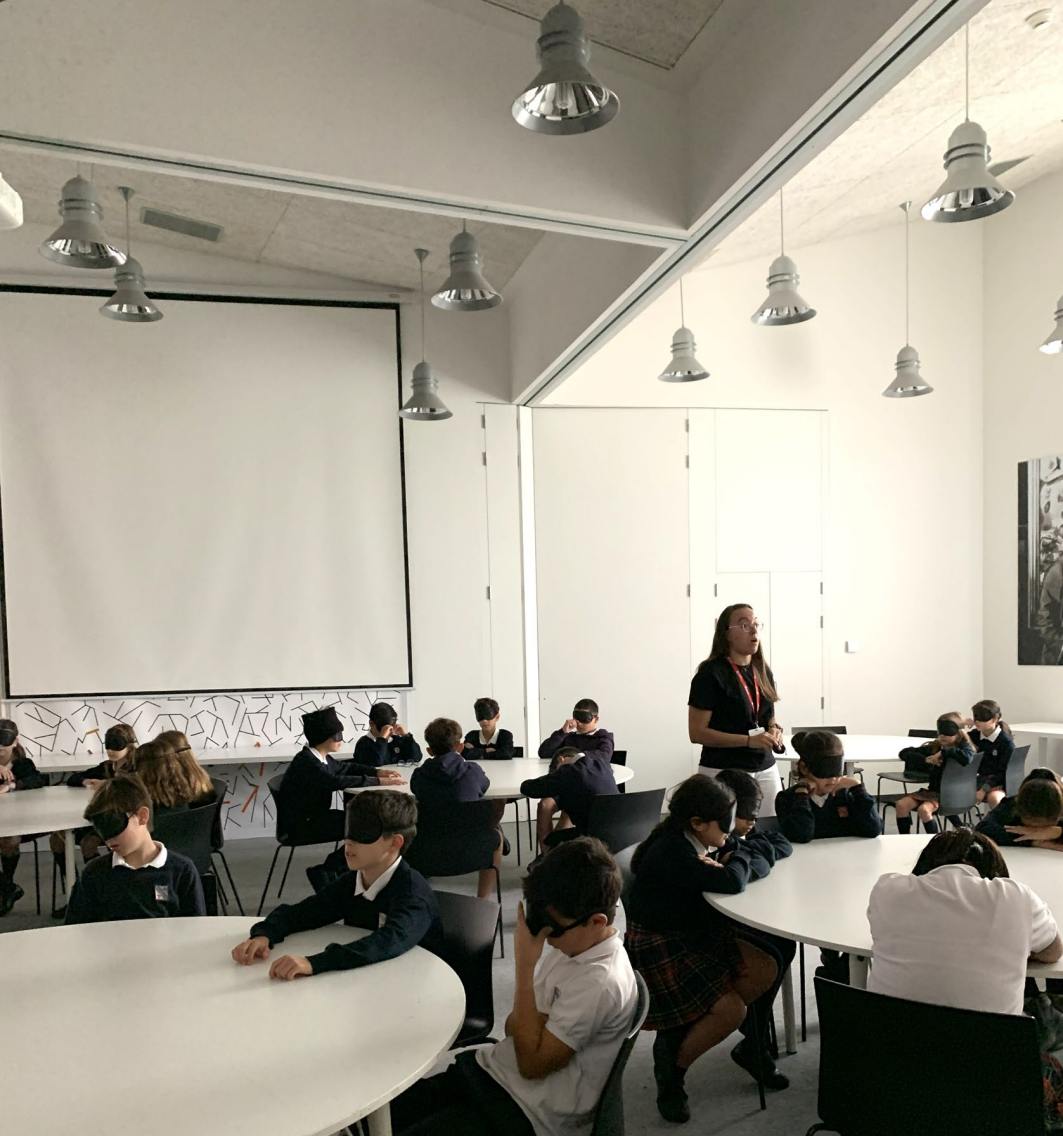
Students with eye masks on.

**Figure 2:**
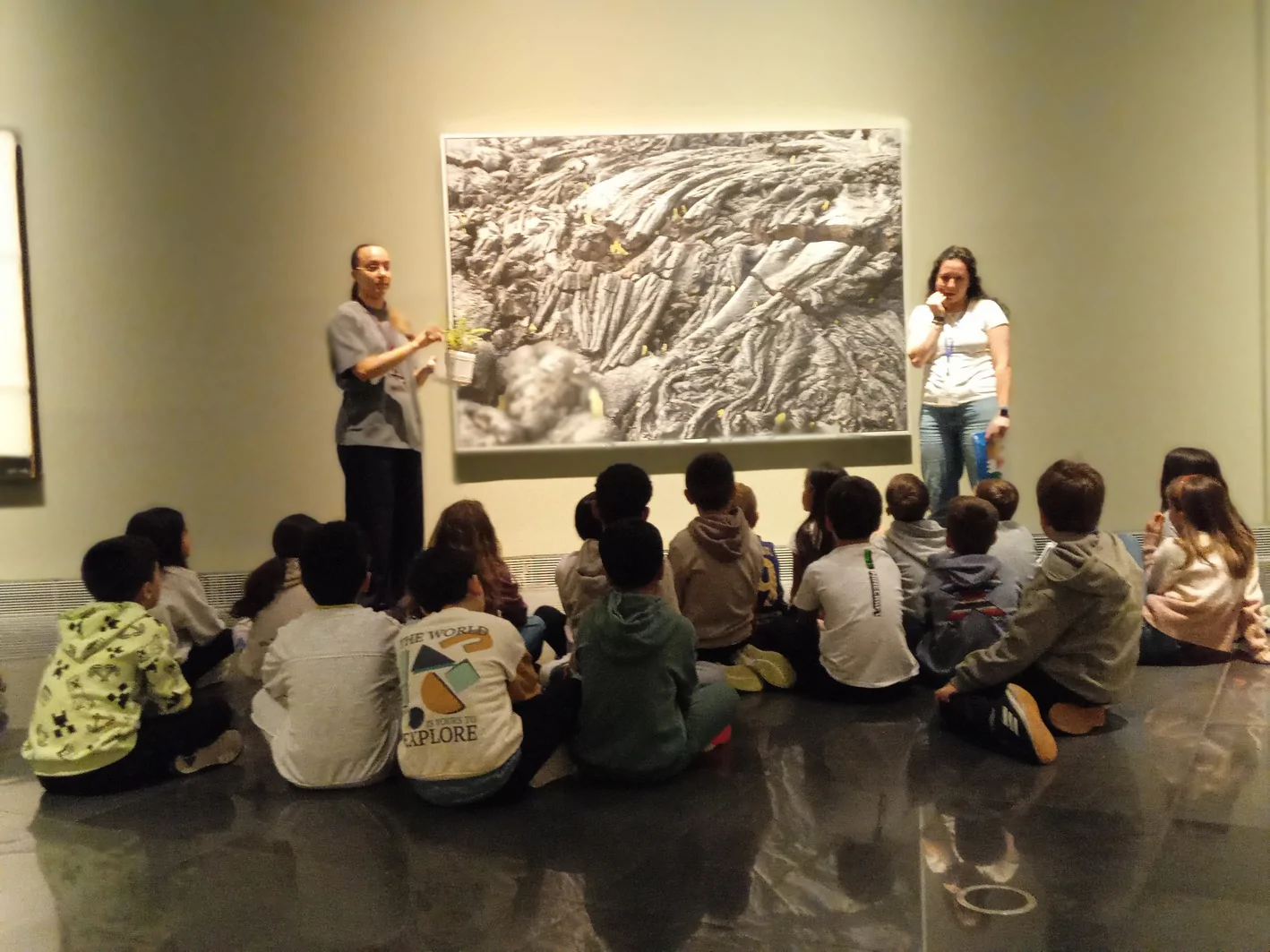
Students observing artworks.

**Figure 3:**
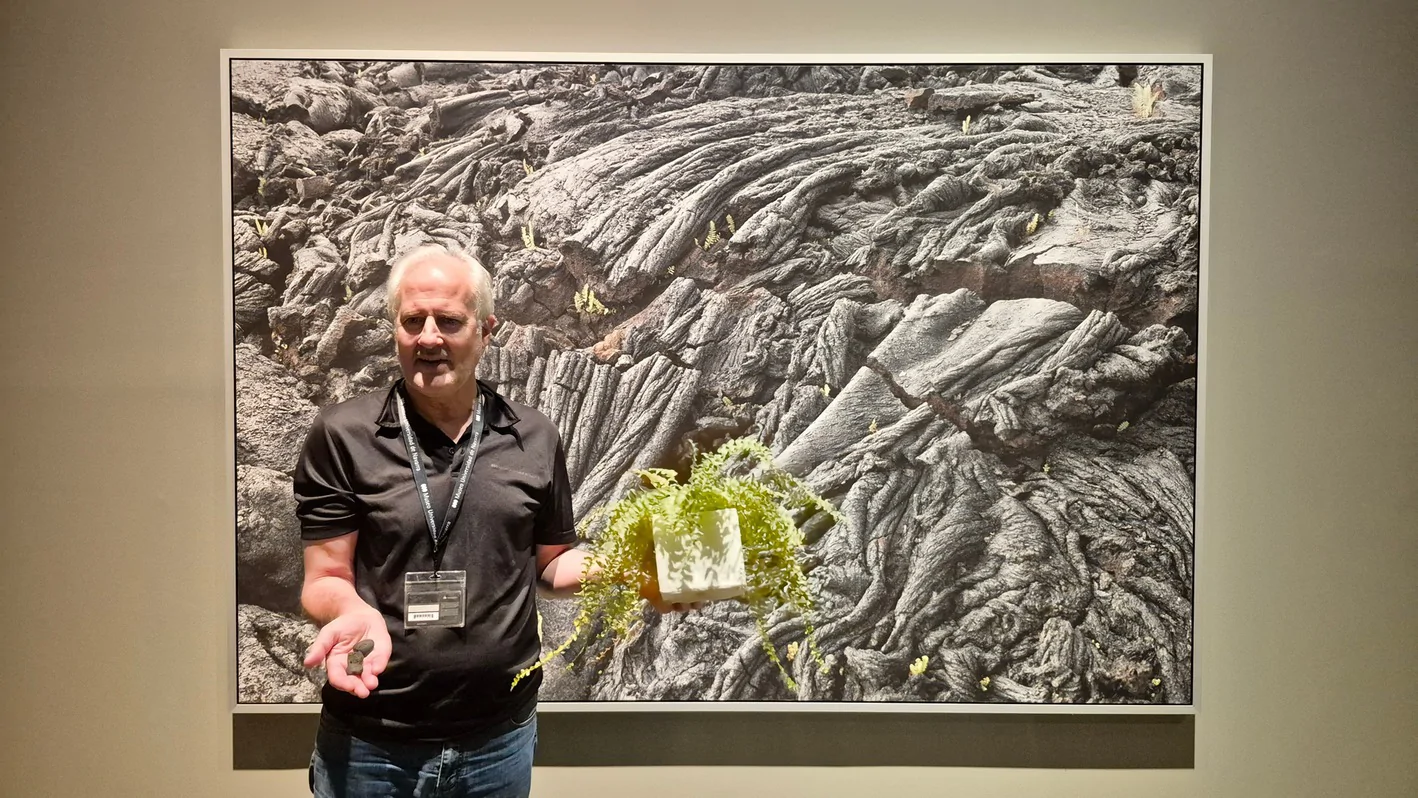
An educator showing a living fern in front of an artwork depicting ferns.

**Figure 4:**
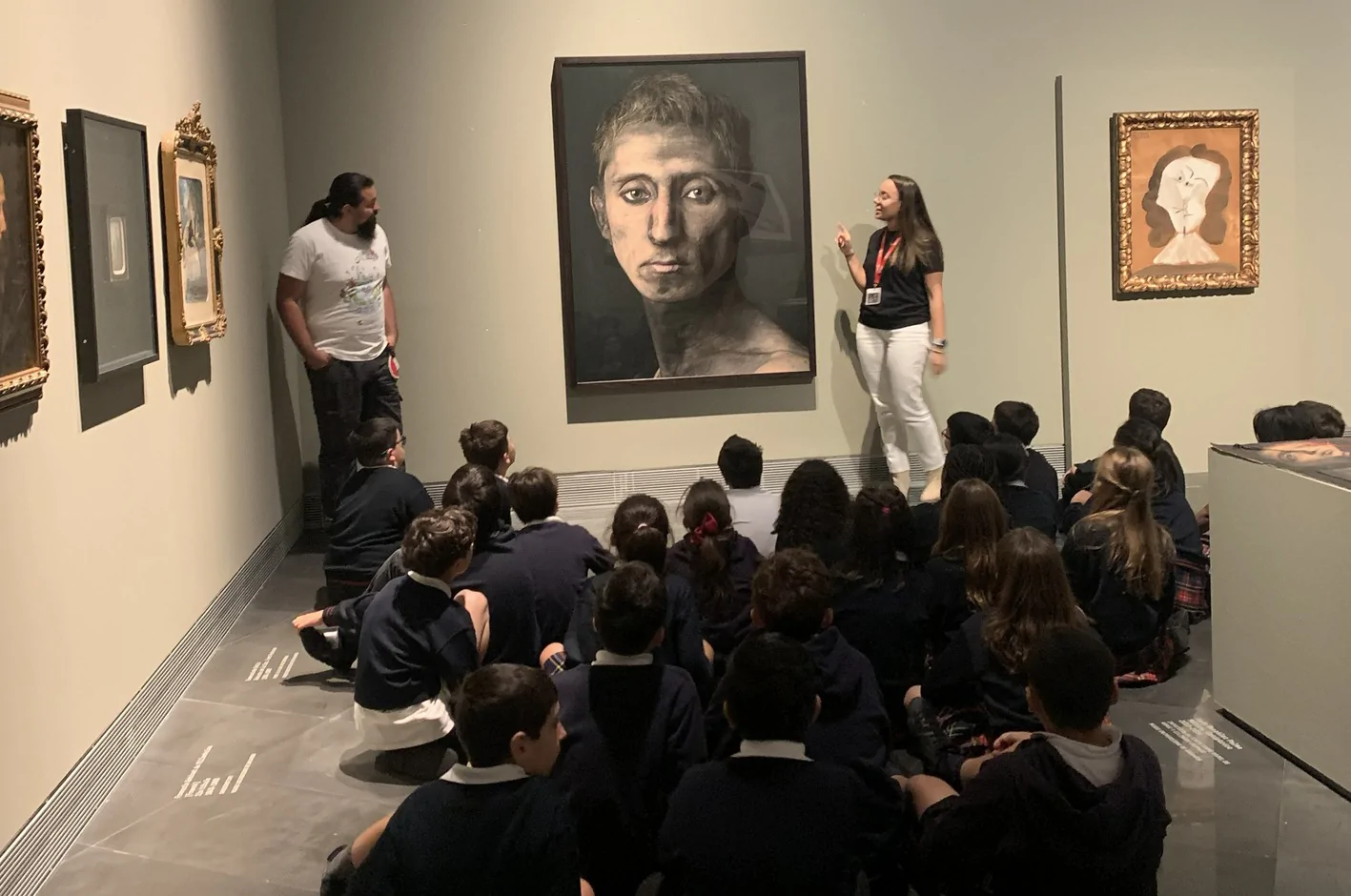
Students engaging with a portrait of a miner.

**Figure 5:**
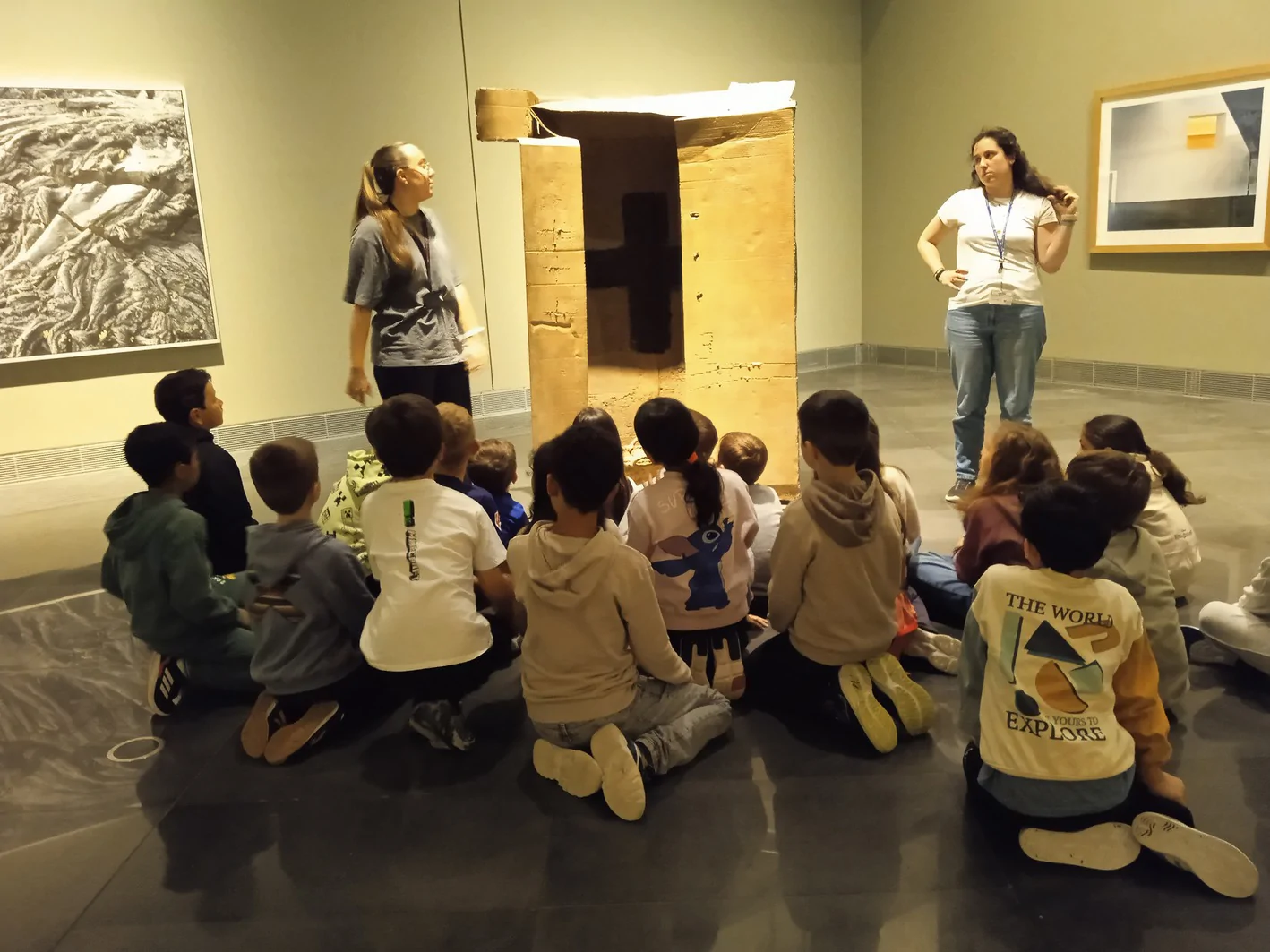
Students observing a work of art about recycling.

**Figure 6:**
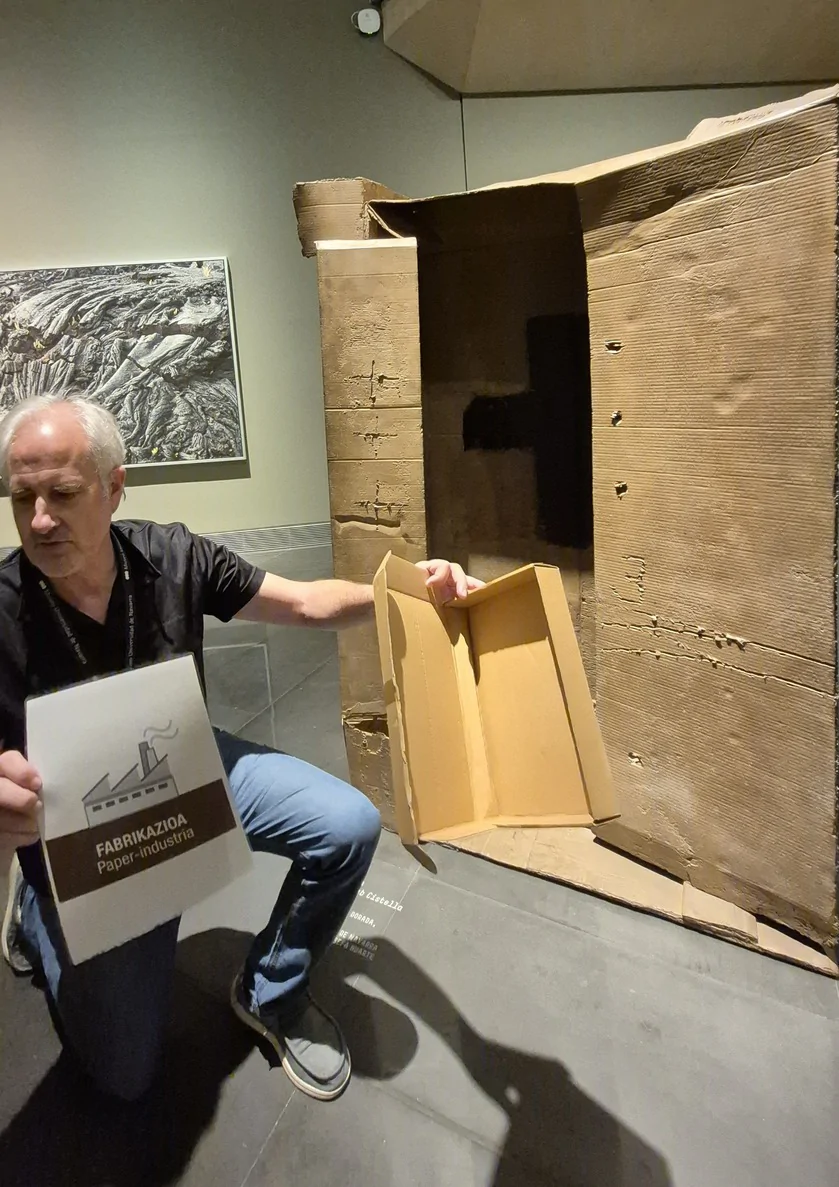
An educator showing a worksheet about recycling and cardboard material.

The most innovative aspects of the activities developed within the program are presented below:

- *Theme*. Deep Ecology is not a common topic addressed in EE programs aimed at school audiences. A program specifically focused on this theme had never previously been implemented within Mancoeduca.
- *Contemporary art museum*. Working on EE in a contemporary art museum, through the artworks it houses, is not a frequent practice.
- *Use of open-ended questions in front of artworks: Visual thinking* (Yenawine, 2013). This approach is used to promote the competencies of learning to think and learning to feel, in order to foster meaningful learning through environmental commitments, thus completing the “feeling–thinking–action” sequence (Novak, 1976).
- *Educational Team*. One of the most innovative aspects of the program was the creation of a joint educational team for its development, composed of art museum educators (MUN) and environmental educators (Mancoeduca). In this way, it was ensured that both environmental and artistic perspectives were effectively integrated into the activities. The joint training of the teams from both organizations during the preparatory phase of the visit helped to avoid discomfort and insecurity among educators when entering areas outside their usual professional practice.
- *Activity A2*. Two exercises related to the education of perception—both internal and external—were carried out. In the first exercise, participants were invited to work in pairs and perform a careful “scan” of their partner, standing face to face. Both participants then identified the small changes introduced in their initial appearance by each member of the pair. The second exercise was individual. Each participant sat on a chair and, with the help of an eye mask, engaged in a breathing and introspective practice in which they explored their personal connection with the natural world (Figure 1).
- *Activity A3*. During the visit to the exhibition rooms, the artwork *Pahoehoe 2* (2018) by the artist Carlos Irijalba (Figures 2, 3) was presented. In front of this photograph, depicting a series of small ferns beginning to colonize the ground after a volcanic eruption, reflection was encouraged on living and non-living beings and their needs. To foster a deeper connection with the artwork, participants were provided with real elements to be sensed and touched, such as a living fern and a volcanic stone. The activity aimed to promote reflection around the question “What do we need to live?”, identifying essential elements for life as well as those that are not fundamental—or are less apparent—but help us establish a connection with the natural world.
- *Activity A4*. The other artwork used was the portrait of a miner titled *Armando II* (2014) by the artist Pierre Gonnord. This piece was used to address themes related to the transformation and exploitation of nature, also alluding to the exploitation of human beings. The finiteness of natural resources and the importance of responsible action and care for the planet were emphasized (Figure 4). In this and other artworks, laminated cards were used to introduce specific concepts such as respect in the extraction of earth resources and the reduction of unnecessary consumption.
- *Activity A5*. In front of the sculpture *Composició amb cistella* (1996) by Antoni Tàpies—a box that appears to be made of cardboard but is in fact bronze—the life cycle of paper and the importance of reuse and waste separation were discussed (Figures 5, 6). A reflection was also made on the concept of cycles, including biological and natural cycles, and the interconnection between all beings.

**Figure 7:**
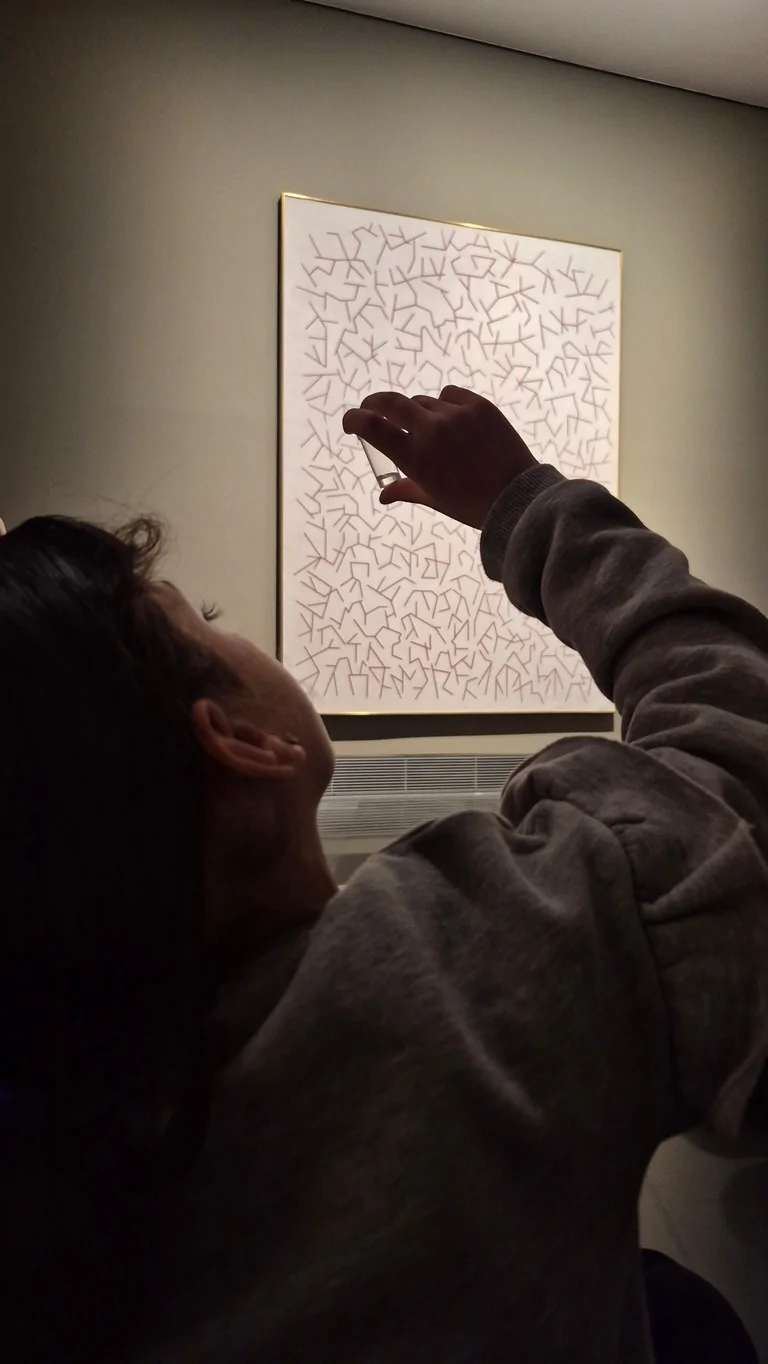
A student looking at a water droplet as part of an artwork.

**Figure 8:**
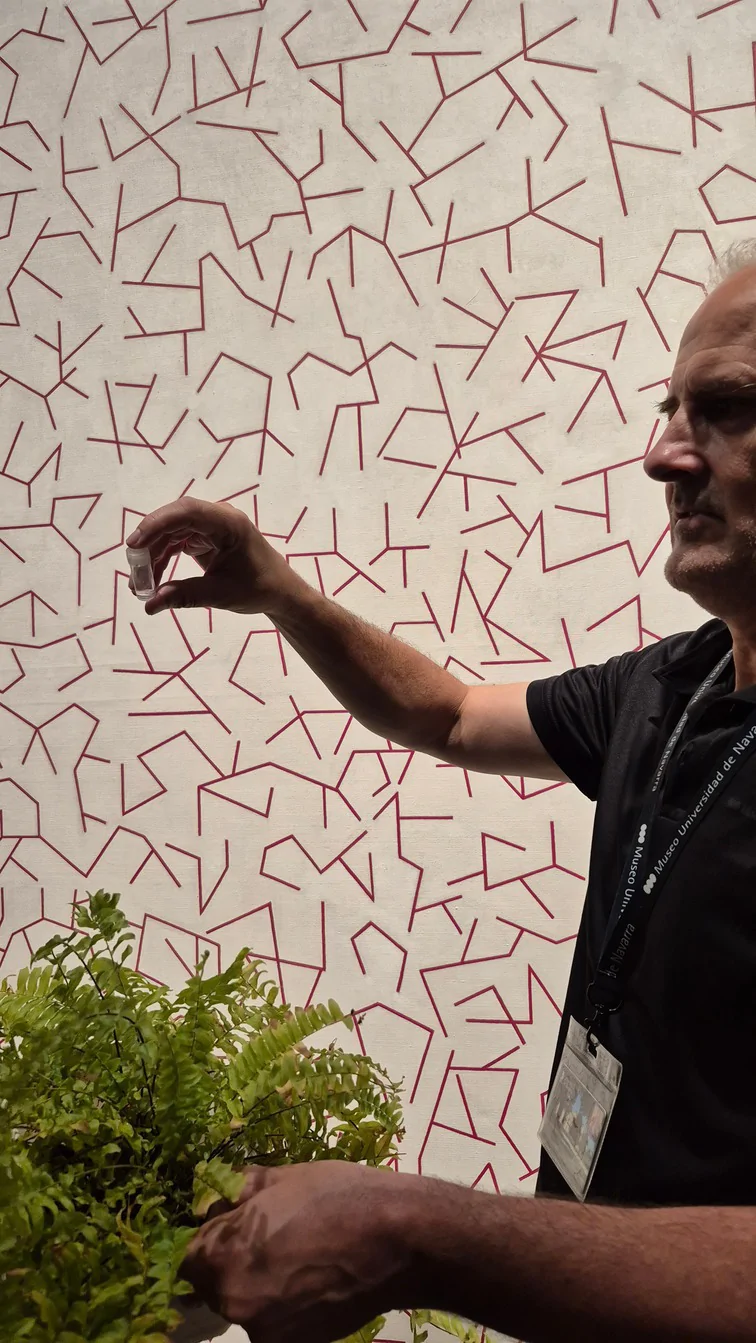
An educator showing the relationship between water and plants.

**Figure 9:**
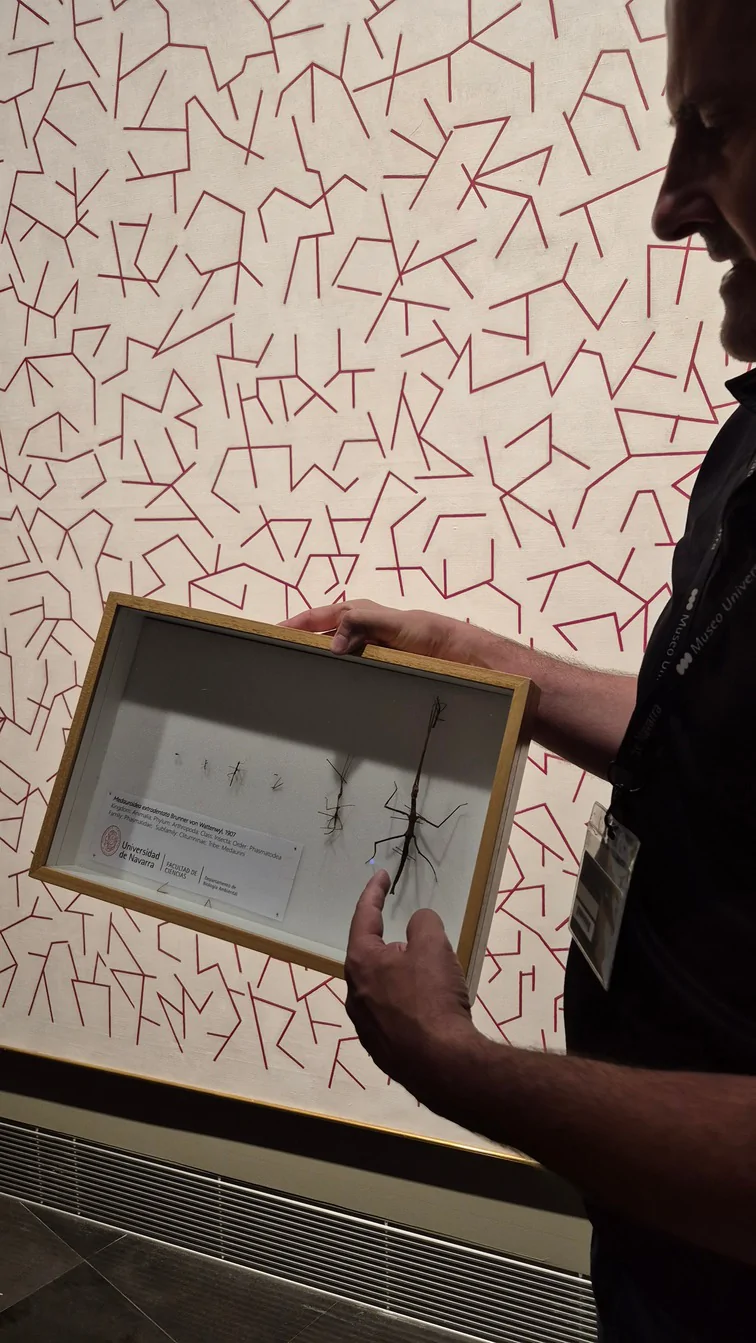
An educator showing the life cycle of an insect and its relationship with water and plants.

The artwork *El número y las aguas I* (2007) by Pablo Palazuelo was also visited. In this work, the water cycle was addressed, and each student carefully observed a drop of water inside a small tube while a poem by Father Damián de Aoiz about the water cycle was read aloud (Figures 7–9), as well as the relationship between water and living beings, both animals and plants. In this way, an additional artistic discipline was incorporated into the activities: poetry.

**Figure 10:**
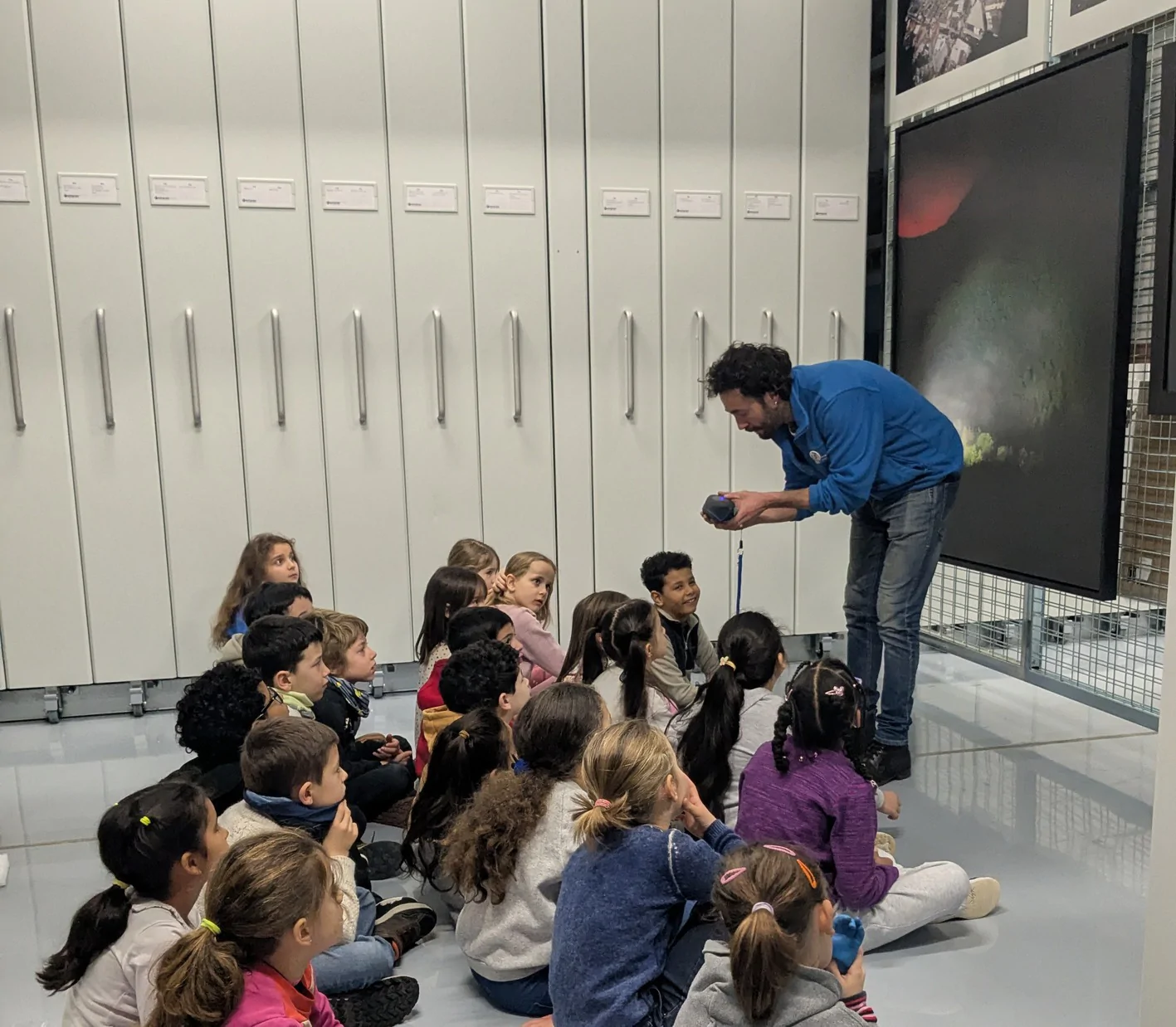
Students listening to sounds of forest animals in front of an artwork.

Finally, in the museum storage areas, another work by Carlos Irijalba was visited, this time *Twilight 10* (2009). In this twilight scene of the Irati Forest (Navarra), the Navarrese forest is partially illuminated by a large artificial spotlight. This image was used to distinguish between diurnal and nocturnal animals, discussing how light pollution affects different living beings within an ecosystem. Special emphasis was placed on nocturnal animals present in this natural environment, viewing images of them and attempting to identify them through their sounds, which were played using a digital audio device (Figure 10).

**Figure 11:**
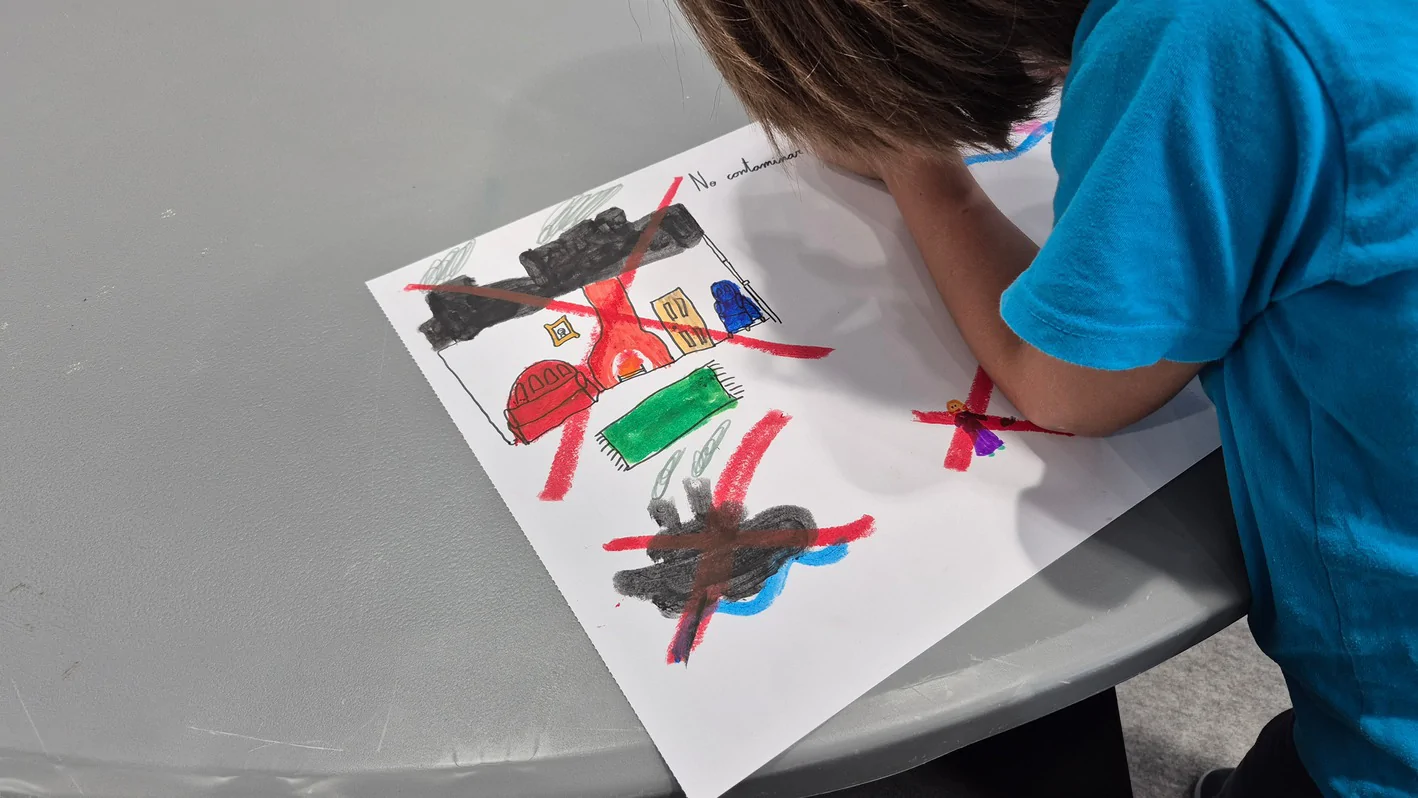
A student making an environmental commitment.

- *Activity A6*. Subsequently, in the museum workshops and through a free creative activity, students were invited to visually represent between one and three individual and concrete commitments to be put into practice in order to promote greater environmental care. In this way, emphasis was placed on action and personal responsibility, fostering students’ empowerment and sense of agency (Figure 11).
- *Activity A7*. Alongside the sharing of the works produced, a collective action was carried out in which the drops of water observed by each participant in Activity A5 were gathered in a single container to water the living fern used in Activity A3, thereby supporting its life. In this way, the exchange between plants and human beings was emphasized, highlighting how humans benefit from oxygen and sugar (glucose) provided by plants. It also illustrated how “every drop makes the ocean,” emphasizing that all individual actions are necessary for environmental care and are potentially powerful and transformative, both individually and socially, at local and global levels.

### 4.4 Target audience and participation

The educational action *EcoArt: A Deep Ecology Adventure in an Art Museum* was offered during the 2025–2026 academic year to school audiences in Primary Education (6–11 years old) and Secondary Education (12–16 years old). It was scheduled every Monday throughout the school year during morning hours. Given the wide age range and the different developmental stages involved, the activity was adapted to be both motivating and enriching for all educational levels and grades.

Participation in the program was very high, with all available sessions fully booked (61 groups and 1.360 students) and more than 500 students placed on a waiting list, making this training action the most demanded among the programs offered by Mancoeduca. This was unexpected, as Mancoeduca already has many well-established educational programs among school audiences, which indicates the strong interest generated by the proposal of integrating Deep Ecology within an art museum context. This does not affect the effectiveness of the program once implemented, but it does provide information about the popularity that this Arts Integration program and EE has had among the teaching staff. Table 3 presents the number of participating groups and students according to educational level.

**Table 3:** Number of groups and number of participating schoolchildren, according to their educational level.

Academic year 2025–2026	Number of school groups	Number of schoolchildren
Primary 1	9	176
Primary 2	9	205
Primary 3	9	211
Primary 4	5	132
Primary 5	11	227
Primary 6	15	339
Secondary 4	3	70
Total	61	1.360

## 5 Results

After the implementation of the educational program, a questionnaire was sent to the 61 teachers of the participating groups in order to assess several aspects related to the development of the program and Deep Ecology. The survey was distributed via Google Forms. The questionnaire contained 27 Likert-type items rated on a 0–5 scale, where 0 corresponded to “Nothing” and 5 to “Very much.” It also included a final open-ended question (Question 28). A total of 17 completed questionnaires were received (28% of participating groups). Table 4 presents the main results obtained.

**Table 4:** Main results obtained.

Question number	Average (max 5)
1. How do you assess the activities as a whole?	4.4
4. Do you think that the didactic methodology of the workshop favours the interest of the students?	4.2
11. What value do you assign to reflection on visual perception to understand ecological issues?	4.4
12. What value do you assign to reflection on the role of emotions in understanding ecological issues?	4.3
17. Do you think that the activities carried out promote the understanding of the fundamental equality between humans, animals and natural elements within an ecosystem?	4.0
18. Do you think that the set of activities managed to make students perceive that all living and non-living beings have an intrinsic value, regardless of their usefulness for humans?	4.0
20. Do you consider that having addressed content on the ecosystem such as climate, atmosphere, soil, water, plants, animals and human beings in the activities has contributed to the development of greater ecological awareness in the students?	4.0
22. Regarding the dialogue generated around the works, did it enrich the group’s learning?	4.2
23. Do you think it is appropriate for students to make specific commitments to the environment?	4.6
24. How pertinent do you find the proposal to carry out artistic-plastic creativity activities to express the link with nature?	4.6
27. How do you assess the possible usefulness of the activities carried out to promote in students a responsible, deep or lasting commitment to the environment?	4.0

### 5.1 Overall evaluation of the EcoArt program (RQ1)

The first research question examined teachers’ overall evaluation of the EcoArt program. Generally, participants expressed a highly positive assessment of the educational intervention, assigning the program a mean overall score of 4.4 out of 5 (Q1).

Teachers considered that the program successfully integrated EE with artistic learning (Q3; M = 4.3) and promoted students’ interest (Q4; M = 4.2), active participation (Q5; M = 4.2) and learning (Q6; M = 4.1). Likewise, the introductory activities were perceived as both clear (Q13; M = 4.2) and motivating (Q14; M = 4.18), while the program was considered appropriate for the educational level of participating students (Q9; M = 4.1).

The qualitative comments reinforce these quantitative findings. Teachers repeatedly highlighted the overall educational quality of the program. One participant described the workshop as “*fully achieving its intended objectives, with students thoroughly enjoying the experience*” (Form 13, Q28), while another emphasised being “*very satisfied with the experience and with the quality of the educational program*” (Form 2, Q28). Together, these comments suggest that teachers perceived EcoArt as a coherent interdisciplinary educational intervention capable of integrating EE, artistic learning and museum education within a meaningful educational experience.

### 5.2 Perceived educational strengths of the program (RQ2)

The second research question explored which educational dimensions of the EcoArt program were perceived by teachers as its principal strengths. The highest-rated aspect of the program concerned the opportunity for students to engage in creative artistic activities to express their relationship with nature (Q24; M = 4.7). Teachers also assigned very high ratings to the activity encouraging students to formulate personal environmental commitments (Q23; M = 4.6), indicating that they regarded this component as particularly effective in connecting environmental reflection with individual responsibility.

Other highly valued dimensions included reflecting on visual perception as a means of understanding environmental issues (Q11; M = 4.4), the final reflection on environmental commitments (Q25; M = 4.4), students’ interaction with the artworks (Q21; M = 3.8) and the dialogue generated around them (Q22; M = 4.1).

Teachers’ comments provide further insight into these evaluations. Several participants highlighted the coherence between the objectives of the program and its educational activities. One teacher particularly appreciated “*the organisation and overall structure of the program*” (Form 13, Q16). Others suggested that the artistic workshop could become even more distinctive by incorporating creative experiences that students would not normally encounter in school. As one teacher observed, “*the workshop could be further developed by introducing materials and artistic activities that students do not usually experience in the classroom*” (Form 8, Q28), while another proposed “*using more engaging artistic materials that students would not normally use at school*” (Form 9, Q28).

Taken together, the quantitative ratings and qualitative comments indicate that teachers particularly valued those educational components that combined aesthetic experience, creative participation, environmental reflection and active student engagement.

### 5.3 Teachers’ recommendations for future implementations (RQ3)

The third research question examined teachers’ recommendations for improving future implementations of the EcoArt program. Analysis of the responses to the open-ended questions (Q16 and Q28) revealed three recurring themes.

The first, and most frequently mentioned, concerned extending the duration of the workshop. Several teachers considered that students would benefit from having additional time for artistic creation and discussion. Participants suggested “*devoting a little more time to the artistic activity*” (Form 3, Q16), “*allowing slightly more time for the workshop*” (Form 12, Q28) and “*extending the practical component of the workshop*” (Form 17, Q28).

A second recurring recommendation involved expanding the museum experience by incorporating a larger number of artworks into the educational itinerary. One teacher commented that “*it would have been nice to see a few more artworks; we were left wanting to explore more*” (Form 3, Q28), while another suggested “*including more artworks located closer to the workshop space together with additional time for the practical activities*” (Form 1, Q28).

Finally, several participants recommended enriching the artistic workshop itself by providing more diverse materials and creative opportunities. One teacher proposed “*incorporating more hands-on materials, such as recycled objects*” (Form 14, Q16), whereas another observed that “*the drawing activity felt somewhat limited because it was something students could easily do in their regular classroom; being in a museum offered an opportunity for a more distinctive artistic experience*” (Form 16, Q28).

Importantly, these suggestions were consistently presented as opportunities to strengthen the educational program rather than as criticisms of its overall quality. Taken together, the findings indicate that teachers perceived EcoArt as a valuable interdisciplinary educational experience while identifying practical improvements that could further enhance future implementations.

## 6 Discussion

The purpose of this evaluative case study was to examine teachers’ perceptions of the EcoArt program as an interdisciplinary educational intervention integrating EE, Deep Ecology, Arts Integration and museum education. Overall, teachers evaluated the program very positively, highlighting its educational coherence, the successful integration of artistic and environmental learning, and the opportunities it provided for students to engage creatively with environmental issues. These findings are discussed below in relation to the existing literature.

### 6.1 Interdisciplinary learning through Arts Integration

The first major finding concerns teachers’ overall evaluation of the interdisciplinary design of the EcoArt program. Teachers not only rated the program highly overall (Q1), but also positively evaluated its capacity to integrate EE with artistic learning (Q3), stimulate students’ interest (Q4), and promote active participation throughout the educational experience (Q5).

These findings support previous research suggesting that Arts Integration provides a pedagogical framework capable of connecting different areas of knowledge through meaningful learning experiences (Burnaford, 2007; Silverstein and Layne, 2010; Goering et al., 2023). Rather than presenting artistic activities as complementary or decorative elements, teachers perceived the artistic dimension as an integral component of environmental learning.

The qualitative comments reinforce this interpretation. Teachers repeatedly described the program as educationally coherent and well aligned with its objectives, suggesting that the integration of artistic interpretation, environmental reflection and museum learning was perceived as a consistent educational proposal rather than as the juxtaposition of different activities.

These findings are particularly relevant because one of the principal challenges identified in EE concerns the need to move beyond disciplinary fragmentation toward educational approaches capable of integrating cognitive, emotional and experiential dimensions of learning (Sterling, 2001; Sauvé, 2005). Teachers’ evaluations suggest that EcoArt constitutes one possible example of such interdisciplinary integration.

### 6.2 Aesthetic experience as a catalyst for environmental reflection

A second important finding concerns the educational value attributed to those activities that combined artistic creation with environmental reflection. The highest-rated components of the program were those inviting students to express their relationship with nature through artistic creation (Q24) and to formulate personal environmental commitments (Q23). Likewise, teachers highly valued activities encouraging visual observation (Q11) and collective reflection on environmental responsibility (Q25).

These findings are consistent with previous studies suggesting that aesthetic experience may contribute to EE by encouraging learners to engage emotionally, imaginatively and reflectively with environmental issues (Inwood, 2008, 2010; Inwood and Kennedy, 2020). Rather than functioning merely as an artistic exercise, creative participation appears to have provided students with opportunities to construct personal meanings around their relationship with the natural world.

The teachers’ recommendations further support this interpretation. Rather than questioning the educational approach itself, participants consistently requested more time for artistic creation and discussion, together with richer artistic materials and experiences. These suggestions indicate that teachers perceived artistic engagement as one of the program’s principal educational strengths and wished to expand, rather than reduce, its role within the intervention.

Taken together, these findings suggest that Arts Integration may contribute to EE not simply by making learning more engaging, but by creating educational conditions that facilitate reflection, dialogue and personal environmental meaning-making.

### 6.3 The educational role of the museum

The findings also highlight the educational contribution of the museum as more than a physical venue for the intervention. Teachers positively evaluated students’ interaction with the artworks (Q21) and the dialogue generated around them (Q22), while several participants recommended extending the museum visit by incorporating additional artworks into future editions of the program. These recommendations are particularly significant because they suggest that teachers viewed direct engagement with artworks as an essential component of the educational experience rather than as a preliminary activity preceding the workshop.

This interpretation is consistent with constructivist approaches to museum learning, which describe museums as environments where knowledge emerges through observation, dialogue and personal meaning-making (Hein, 1998; Falk and Dierking, 2016; Hooper-Greenhill, 2007). Similarly, Dewey (1934) argued that aesthetic experience becomes educational when learners actively engage with works of art through perception, interpretation and reflection. Within EcoArt, the artworks functioned as mediating objects that encouraged students to examine environmental issues from new perspectives. Teachers’ recommendations to include additional artworks therefore reinforce the educational value attributed to the museum experience itself.

### 6.4 Educational implications

Several educational implications emerge from these findings.

First, teachers’ evaluations suggest that interdisciplinary collaboration between environmental educators and museum educators can generate coherent educational experiences capable of integrating environmental learning with artistic interpretation.

Second, the findings support previous research highlighting the educational potential of museums as environments that promote observation, dialogue and reflective learning. Rather than functioning simply as venues for educational activities, museums may contribute actively to the development of environmental awareness by providing contexts in which aesthetic experience supports environmental reflection.

Finally, the findings lend empirical support to the conceptual framework underpinning the EcoArt program. In this framework, Deep Ecology provides the environmental philosophy, Arts Integration constitutes the pedagogical approach, and the museum offers the experiential learning context. Teachers’ evaluations suggest that these three dimensions operate in a complementary manner, generating educational experiences perceived as coherent, engaging and meaningful.

Although the present study does not evaluate changes in students’ environmental behaviour, it provides evidence that teachers perceive this interdisciplinary framework as educationally valuable and worthy of further development.

### 6.5 Ethical considerations

Ethical approval for the study, along with the consent form and questionnaires, was granted by the University of Navarra Ethics Committee (reference number: project 2025.268; approval date: 28/11/2025). Prior to data collection, participants were informed about the voluntary and anonymous nature of their participation in the study. The University of Navarra Ethics Committee confirmed that the information and consent process complied with the legal requirements of Spanish law. Research transparency is a core principle of this study. Consequently, all activities related to the design, planning, and implementation of the study have been conducted in compliance with the Spanish Protection of Personal Data and Digital Rights Act (LOPD) 3/2018. The content management tool provided by the University of Navarra was used to ensure the ethical collection and storage of all research data, in accordance with the European Data Protection Regulation (GDPR 2016/679).

## 7 Limitations

This study had several limitations. The study evaluates teachers’ perceptions rather than students’ learning outcomes. The response rate from the teachers sample to the questionnaire has been low. It would have been better to have more responses from the participating teachers. The study does not include comparative studies between groups nor longitudinal studies.

The study assessed an educational intervention of relatively short duration. The very dynamics of school visits to museums involve several difficulties, including transportation and the duration of the programs that take place there. Although extending the duration of the program is one of the teachers’ suggestions, it is not easy to increase the time dedicated to the program.

## 8 Conclusion

EcoArt program may offer an example of how Environmental Education, Arts Integration and museum education can be brought together within a coherent educational framework. This program shows that Arts Integration could provide a valuable pedagogical approach through which environmental philosophy may be translated into meaningful educational experiences. Within the EcoArt program, Deep Ecology, Arts Integration and museum education appear to complement one another in ways that teachers perceived as coherent, engaging and educationally meaningful.

School teachers can perceive EcoArt as a valuable program that may successfully combine artistic engagement with environmental learning within the context of an art museum. The study suggests that collaboration between schools, museums educators and environmental educators may offer promising opportunities for designing interdisciplinary educational experiences. It would be advisable to continue implementing this program in order to incorporate the different suggestions provided by teachers. It would even be beneficial to develop more Deep Ecology programs in art museums to further explore the potential for environmental awareness and agency among school audiences through the meaningful experiences that such initiatives are capable of providing.

Taken together, the findings suggest that EcoArt may represent a promising example of interdisciplinary collaboration between Environmental Education, Arts Integration and museum education. Although this study was limited to teachers’ perceptions within a single educational program, it provides empirical evidence that this type of educational intervention is perceived as coherent, relevant and worthy of further development.

Future research should examine students’ own perspectives, explore whether similar findings emerge in different educational and cultural contexts, and investigate the long-term educational impact of interdisciplinary museum-based programs. In particular, further studies could analyse whether educational experiences such as EcoArt contribute to fostering environmental awareness, agency and meaningful relationships with the natural world over time.

This study suggests that integrating Environmental Education, Arts Integration and museum education may constitute a valuable educational pathway for designing learning experiences that connect aesthetic engagement with environmental reflection. While additional empirical evidence is needed, the EcoArt program provides an example of how these educational traditions can be articulated within a coherent interdisciplinary framework.

## Data Availability

The raw data supporting the conclusions of this article will be made available by the authors, without undue reservation.

## Footnotes

1 In the 2024–2025 academic year, Mancoeduca served 1,831 school groups, with a total of 39,207 participants.
