# Supplementary material for: Integrating arts and Environmental Education in museums: an evaluative case study of the EcoArt program
Source: Front Psychol. 2026 Sep 3;17:1917355. doi: 10.3389/fpsyg.2026.1917355 (PMC13581814; doi:10.3389/fpsyg.2026.1917355)
Supplement: Supplementary file 1 [file Supplementary_file_1.DOCX]

**APPENDIX 1.**

| **EDUCATIONAL ACTION**  *ECOARTE: A deep ecology adventure in an art museum* | |
| --- | --- |
| **TYPE OF ACTIVITY**  Guided tour | **THEME**  No Footprint |
| **TARGET AUDIENCE**  School Primary and Secondary School | **DURATION OF THE ACTIVITY**  2 hours |
| **HUMAN RESOURCES**  1 MUN educator + 1 Mancoeduca educator | **RECOMMENDED NUMBER OF PARTICIPANTS**  Classroom group |
| **OBJECTIVES**  **GENERAL OBJECTIVES**   - To raise awareness through art about the importance of our behaviour on the environment. - Promote environmentally responsible behaviors in a global way through concrete commitments.   **SPECIFIC OBJECTIVES**   - Educating the gaze through art to know the environmental reality in more depth. - Recognize the intrinsic link of each person with nature. | |
| **SDGs**   | |
| **KEY COMPETENCES FOR SCHOOL AUDIENCES (LOMLOE)**  Breakdown by primary cycles in [the teachers' file](https://docs.google.com/document/d/1HsMC0RN7I4GJQgUc5X7swcIAmn-gjpKEFN-G55M855s/edit?tab=t.0) | |
| **CCM STRATEGIC PLAN**  **Axis 2: Relations with citizens and the environment:**   - Challenge 4: Promote the image of MCP/SCPSA based on a commitment to sustainability and Corporate Social Responsibility | |
| **GreenComp** **Scope** \| **Competencies**; Addressing sustainability values \| - Appreciation of sustainability; Embracing the complexity of sustainability \| - Systems thinking - Critical thinking; Acting for sustainability \| - Collective action - Individual initiative | |
| **METHODOLOGIES**  Experiential learning | |
| **NESE ADAPTATIONS**  [**Script for the base_ACCESIBLE_MCP educational team**](https://docs.google.com/document/d/1EquvwTe6UTQpA3qhaBsROKkLZGjxGNyH45_4ABk0sxs/edit?usp=drive_link) | |
| **DESCRIPTION AND DIDACTIC SEQUENCE** Brief description of the activity, the methodology and its timing. Phase \| Time; Introduction \| 5; Motivation \| 15’; Research \| 50; Creative activity \| 35’; Reflection \| 10’; Closure and evaluation \| 5’ | |
| **Presentation of the activity: Welcome and introduction** | Duration: 5' |
|  | Materials: |
| The educational action will be led by two people per group, an educator from the MUN and an educator from Mancoeduca.  We receive the group at the entrance and move to the workshop where they will leave the coats and backpacks and the activity will begin.  In a large group (the two classes together) One of the people on the team introduces the others and the activity.  They are asked open-ended questions to introduce the place where they are (art museum) and what they are going to do. *Do you know where we are? Have you ever been to a museum? Have you been to an art museum?* We are going to see works of art in the rooms. The rules to be complied with are recalled.  They are told that we are going to talk about how to take care of the environment through something very special such as art. We know through our gaze. That is why we are going to exercise it, so that it is attentive. | |
| **Motivation**  **Learning to look**  **Discovering my link with nature** | Duration: 15' |
|  | Materials: 30 fabric masks |
| In a large group, this dynamic will be carried out by the MUN team. The Mancoeduca team will remain attentive and supportive.   - First we look at each other in pairs, performing a "scan" on the partner, and we turn around to make 1 change in our appearance. We turn around to look at each other and try to guess the change that has taken place. - With masks and sitting in chairs, we look inside ourselves and look for our link with nature. We pay attention to our breathing and our body, which is made up of 70% water. We need breath and water to survive. We take deep breaths, breathing in and out slowly.   After these dynamics, the two classes will be separated and each of them will go with a MUN-Mancoeduca couple to the rooms. | |
| **Research**  **Art and the environment** | Duration: 50' |
|  | Materials: Chips/cards, speakers, fern, tubes and stick insect. |
| ORGANIZATION OF THE ITINERARIES BY GROUP ROOMS  The two groups will make the two itineraries simultaneously but in reverse order.  Group **1 (itinerary A+B)**   - They will first go to the room where the works of Irijalba, Tàpies and Gonnord are located (itinerary A) and then they will go to the warehouse and the room with the Palazuelo (itinerary B).   Group **2 (itinerary B+A)**   - you will go down first to the warehouse and then go up to the room to see the Palazuelo (itinerary B) and then to the room where the works of Irijalba, Tàpies and Gonnord (itinerary A) are located.   The duration of the two itineraries will be similar.  The concepts that we will discuss in theaters will be:   - Different views of the environment - Introduction to the concept of deep ecology: equality between human beings and other natural beings (non-exploitation, minimization of ecological footprint...)   **ROUTE A (20 ́)**  **Original nature—the human being who transforms it—how to close the circle**    **Explanation of the work:**  Contents: Alive/not wine. Volcanic rock, soil and plant elements (ferns). La Palma Volcano.  **Edu MUN:** This photograph is by a Navarrese artist, Carlos Irijalba, whose work we will see later. He is photographing the beginning of life with a look at the importance of soil and caring for nature, the smallest, which has just been born. Later on, other species in this photo will follow one another: insects, beetles, spiders... Other living beings. They are shown a live fern.  **Edu MANCOEDUCA:**  **ORIGINAL NATURE**  Is there life in a volcano? There are microorganisms that can live in rocks and volcanic waters withstanding high temperatures. On this lava flow we see how small fern shoots make their way, how did they manage to grow there? The rain has been able to leave fertile soil, the seeds have been able to arrive in the excrement of a bird, some have germinated and are beginning to grow.  Let's talk about animate and inanimate. rocks and living beings. Depending on the age of the students, the questions may be more or less complex, but we always start from their own experience, from what is close and what is everyday.  What do you need to live? (terms such as water, air, food, sun, perhaps house, clothes, even affection, friends or family will come up) How can that fern survive on the rock? (We deal with the different needs of living beings to survive). That fern doesn't need the same thing as a human being, does it? We show a series of images so that they can choose elements that are essential for the life of natural beings and human beings (food, water, clothing, shelter or house, sun...) and also other elements that are not so necessary but without which we no longer know how to live (energy to move a car, electric light, etc.). a mobile phone...) What are these elements made of? Where are they extracted from? How long do they last? This will help us to talk about the extraction and exploitation of resources by introducing the following work.    **Explanation of the work:**  Contents: Exploitation of natural resources, minerals. Living and non-living beings. Effort, energy, interaction between people and nature in a sustainable way, looking towards nature and towards people.  Pierre Gonnord was a French-born photographer who died in 2024 and looked deeply at people in their contact with nature through his professional activities. He worked with disadvantaged groups such as gypsies or mining immigrants. He was called "the photographer of the soul". We are going to look at this portrait carefully, just as we have done in the workshop to find out the changes. He also gives us a deep look.  Questions: How are you? What does he want to tell us? What emotions does your gaze convey?  **What does exploitation and exploitation mean?** We asked the whole group if they know what exploitation and exploitation mean. The older ones may know it, but the little ones probably don't and we can stay talking about emotions.  *We agree that there is no time to do the following dynamics, but its conclusions can be mentioned:  We distributed cards among the group with different words and explanations of the term "exploit" such as, for example, "action and effect of exploiting, extracting from the mines the wealth they contain, making a profit from a business or a company, abusively using the work of another person for our benefit, exploitation, benefit, use, obtaining, abuse, deception, factory, company, installation, industry...". Looking at the photograph, we choose what we believe to be explanations of the words (they all are) and we reflect on what is the miner exploiting? Is he being exploited? What emotions does his face express? What does it make you feel? If planet Earth had feelings, how would it feel when we extract materials from it? Would you be happy to share and help us? But what if you go too far? We dealt with the issue of scarcity and overexploitation of natural resources.  With ESO we will work on [Chief Seattle's letter](http://herzog.economia.unam.mx/profesores/blopez/valoracion-swamish.pdf)    **Explanation of the work:**  Contents: Authenticity and appearance, recycling, reuse, reduction, responsibility and care, material cycle, individual commitments. Containers. What to do so as not to overexploit.  Antoni Tàpies created artistic objects with "poor" materials, apparently worthless. He plays with our perception and our prejudices by making a hyper-realistic box that looks like cardboard but, in reality, is made of bronze and weighs more than 600 kilos.  **Use and throw away or use and recycle?** We show a real cardboard box, what is the difference between our box and this one from Tàpies?, which lasts longer?, which is more functional for our daily life? We provide them with different cards with the cycle of a paper box (tree, pulp mill, cardboard processing, cardboard becomes a box, we use the box, we deposit it in the paper and cardboard container and it is made into cellulose pulp again). It can be a set of cards for the whole group or several and make subgroups. We ask them, together, to assemble it in order and we will correct it. We show the linear cycle (each person with their card in a line), with the trees, and then remove them and make it circular (we put the people with their cards in a circle to show it. We reflect, What will be better for nature? What will be best for us?  **ROUTE B (20')**    Before arriving, we explain to them that they are going to visit a very special place, which is inside the earth, we are going to go down, to the underground. It is a place that cannot normally be entered, where the secrets of the museum are kept: the sala de peines (warehouse of works). We warn you that  Contents: Light pollution, forests, human/nature interaction, wild nightlife.  **Explanation of the work:** Carlos Irijalba is an artist from Pamplona who, since he was a child, already had a different view of the world, an artist's gaze. Let's look carefully at this work to find out if there is anything strange in the image, if something catches your attention. Irijalba seeks to know reality in a different way, through large-scale artificial light, where there is no light.  **When does nature sleep?** After listening to the explanation about the work and once they have observed it carefully, we will ask the group to name us, if they know, more animals that can live there (we will point out that it is Irati, which is a little while by car from our houses, no lions or crocodiles live there..) We will distribute photographs of bats, fireflies, owls, owls, toads and moths. These are some of the animals that live in that forest but that we cannot see in the photo. With a loudspeaker, we will put different sounds so that the students can tell us which animal they think they are (taking the photos) Have you ever heard it? Where were you when you heard it? We return to the animals that have been named before to give notions of nocturnal and diurnal animals (we show a file) What happens if a light is turned on in the forest? What will those animals that have been awakened feel? And those who wanted darkness and are dazzled? (We can make a comparison with when we are sleeping and someone turns on the light). What will happen to animals living in cities? Will their cycles be the same as in nature?    **Explanation about the work:**  Contents: Social diversity, biodiversity, ecosystem, water, water pollution, potential for change when the social is ordered.  Pablo Palazuelo was an abstract artist whose art combines geometry and nature. It tells us about the order and disorder that we find in it. Biology, life is orderly, it seeks order. It needs energy to organize itself. Entropy: matter tends to disorder. But energy recomposes it. Discover the geometry/order of things. We are the way we are because there is the order of the natural.  **Observe to see. Every drop makes the ocean**. We start with the observation of a drop of water in a closed tube (one per person). While they watch her move the tube, the poetry about the water cycle is read. When we finish reading the poetry, we ask the group what they see and feel. We connect with the basic needs of living beings that we have talked about before. Our body is made up of 70% water, we need it to survive. Uses of water (showing photographs).  Can we drink water directly from nature, for example from rain? We need to treat it, make it drinkable. We look at our drops and now to work, what do they have in common? We look at an individual figure and the whole: like our drops, which individually are small and insignificant but together take on a meaning and make an ocean, in the painting the lines also form figures and a composition.  Let's now look at the stick insect, does it resemble any of the shapes in the painting in front of us? Have we seen any of these insects in the wild? To see them you have to pay attention, they look like parts of the branches and only if we look closely can we see that they are animals and not parts of that plant. Just like in this painting, that only when we look at it we see that each element is different.  If the group has already completed the two itineraries, with the bottles of water drops in hand they will go up to the workshop. If they still have to complete itinerary 1, the educator will put all the jars in a bag and keep them (they are given to the teacher to take them during the visit).  **At the end of the two itineraries they go up to the workshop where they will meet again in a large group.**   - Cortazar text for secondary school | |
| **Action/Communication**  **Creative activity and reflection, critical thinking**  **I am an eco-artist** | Duration: 35' (creative activity) + 10' (reflection) |
|  | Materials: Square cardboard for the first cycle of primary school, leaflets for the second cycle, leaflets for the third cycle and cubes for secondary school. Paints, markers, collage, etc. |
| Upon arriving at the workshop, each student will pour their drop into a spray bottle to signify the sense of the whole, citizenship, in front of the individual. Each of us can do little things that, all together, are very powerful and transformative. With the sprayer we will lightly water our fern, closing the circle of care and connection with nature.   - Artistic activity to represent the link with nature. - Reflection and choice of 1 to 3 personal or group environmental commitments that reduce our environmental impact (propose topics related to CCM: transport, consumption/waste, water, nearby natural environment...). - Sharing of works and commitments. The students will comment on the commitments acquired at tables or in front of the whole class. - The reflection can be by tables or in groups depending on the number of visiting students. The educators will energize this activity and will be in charge of recapitulating the main ideas and making the final reflection and gratitude. - Final reflection: Show them the fern again and water it with the water gathered with all its drops. | |
| **Closure and evaluation** | Duration: 5' |
|  | Materials: |
| - Farewell to the group - Assessment survey | |
| **PRE- AND POST-ACTIVITIES**   - Prior (to be determined by each educational level) - Subsequent (to be determined by each educational level) | |
| **MATERIAL AND LOGISTICAL RESOURCES**  Detail the educational materials necessary for the development of the activity: They are detailed in each section of activities. | |
