# Supplementary material for: Integrating arts and Environmental Education in museums: an evaluative case study of the EcoArt program
Source: Front Psychol. 2026 Sep 3;17:1917355. doi: 10.3389/fpsyg.2026.1917355 (PMC13581814; doi:10.3389/fpsyg.2026.1917355)
Supplement: Supplementary file 2 [file Supplementary_file_2.DOCX]

**APPENDIX 2.**

**Ecoarte Program Assessment Scale: A Deep Ecology Adventure in an Art Museum**

**School:**

**Course:**

**INSTRUCTIONS**

This survey is part of a research project. The objective is to know his assessment of the workshop "Ecoart: An adventure of deep ecology in an art museum".

We want to inform you that this survey is completely anonymous; None of your answers will be linked to your personal identity. The data will be used exclusively for academic and scientific purposes.

Please answer the following questions by ticking the chosen box, as shown in the example of the first Q1 question.

**It is very important, please, that you answer all the questions.**

**Thank you very much**

| **1.** | **On the novelty and fit of the program** | **Nothing**  **1** | **Almost nothing**  **2** | **Little**  **3** | **Quite**  **4** | **Very much**  **5** |
| --- | --- | --- | --- | --- | --- | --- |
| **Q1** | Materials |  |  |  | X |  |
| Q2 | How would you quantify the workshop's innovative approach in relation to the environmental education programs you know about or have been carrying out so far? |  |  |  |  |  |
| Q3 | To what extent do you consider that the environmental approach through art is integrated with the objectives of the school curriculum of your course? |  |  |  |  |  |
| Q4 | Do you think that the didactic methodology of the workshop favors the interest of the students? |  |  |  |  |  |
| Q5 | Do you think that the didactic methodology of the workshop favors the participation of the students? |  |  |  |  |  |
| Q6 | Do you think that the didactic methodology of the workshop favors student learning? |  |  |  |  |  |
| Q7 | Do you think that the didactic methodology of the workshop favours the environmental commitment of the students? |  |  |  |  |  |
| Q8 | What did you think of the structure of the activities? | Not suitable at all |  |  |  | Very suitable |
| Q9 | Do you think that the activities are well suited to the needs of your students? | Not suitable at all |  |  |  | Very suitable |
| Q10 | In your opinion, is the duration of two hours adequate? | Short answer text: | | | | |

| **2.** | **About the introduction and motivation (attentive gaze and personal bond)** | **Nothing**  **1** | **Almost nothing**  **2** | **Little**  **3** | **Quite**  **4** | **Very much**  **5** |
| --- | --- | --- | --- | --- | --- | --- |
| Q11 | What value do you assign to reflection on visual perception to understand ecological issues? |  |  |  |  |  |
| Q12 | What value do you assign to reflection on the role of emotions in understanding ecological issues? |  |  |  |  |  |
| Q13 | Do you think that the initial introduction (dynamics of attentive gaze, breathing, connection with nature) was clear for the students? |  |  |  |  |  |
| Q14 | Do you think that the initial introduction (dynamics of attentive gaze, breathing, connection with nature) was motivating for the students? |  |  |  |  |  |
| Q15 | What do you think was the attitude of the students after the introductory and motivational activities? | Negative |  |  |  | Positive |
| Q16 | Would you include any changes in the introductory activities – motivation (dynamics of attentive gaze, breathing, connection with nature)? If so, indicate which ones. | Long Answer Text: | | | | |

| **3.** | **On research and exploration in rooms (concepts of ecosystem equality, responsibility and non-exploitation)** | **Nothing**  **1** | **Almost nothing**  **2** | **Little**  **3** | **Quite**  **4** | **Very much**  **5** |
| --- | --- | --- | --- | --- | --- | --- |
| Q17 | Do you think that the activities carried out promote the understanding of the fundamental equality between humans, animals and natural elements within an ecosystem? |  |  |  |  |  |
| Q18 | Do you think that the set of activities managed to make the students perceive that all living and non-living beings have an intrinsic value, regardless of their usefulness for humans? |  |  |  |  |  |
| Q19 | Do you think that students have understood the inherent value of nature regardless of its usefulness to human beings? |  |  |  |  |  |
| Q20 | Do you think that having addressed content on the ecosystem such as climate, atmosphere, soil, water, plants, animals and human beings in the activities has contributed to developing a greater ecological awareness in the students regarding the interconnection of the elements of nature? |  |  |  |  |  |
| Q21 | How do you assess the participation and interaction of students with works of art? |  |  |  |  |  |
| Q22 | Regarding the dialogue generated around the works of art, did it enrich the group's learning? |  |  |  |  |  |

| **4.** | **Creative activity and reflection (commitment and action)** | **Nothing**  **1** | **Almost nothing**  **2** | **Little**  **3** | **Quite**  **4** | **Very much**  **5** |
| --- | --- | --- | --- | --- | --- | --- |
| Q23 | Do you think it is appropriate for students to make specific commitments to the environment? |  |  |  |  |  |
| Q24 | How pertinent do you find the proposal to carry out artistic-plastic creativity activities to express the link with nature? |  |  |  |  |  |
| Q25 | From your experience, do you think that the final reflection on environmental commitments consolidates learning? |  |  |  |  |  |
| Q26 | Do you consider that the activities promote the adoption of sustainable practices among students, thus contributing to a harmonious coexistence with the environment through the environmental commitments they assume? |  |  |  |  |  |

| **5.** | **Overall rating** | **Nothing**  **1** | **Almost nothing**  **2** | **Little**  **3** | **Quite**  **4** | **Very much**  **5** |
| --- | --- | --- | --- | --- | --- | --- |
| P27 | How do you assess the possible usefulness of the activities carried out to promote in students a responsible, deep or lasting commitment to the environment? |  |  |  |  |  |
| P28 | What suggestions or improvements would you propose for future editions of the program? | Long Answer Text: | | | | |

We thank you very much for your time and collaboration in conducting this investigative survey. Thank you very much.
